# Biotechnological potential of microbial bio-surfactants, their significance, and diverse applications

**DOI:** 10.1093/femsmc/xtad015

**Published:** 2023-08-10

**Authors:** Renuka Kumari, Lairenjam Paikhomba Singha, Pratyoosh Shukla

**Affiliations:** Enzyme Technology and Protein Bioinformatics Laboratory, School of Biotechnology, Institute of Science, Banaras Hindu University, Varanasi 221005, Uttar Pradesh, India; Enzyme Technology and Protein Bioinformatics Laboratory, School of Biotechnology, Institute of Science, Banaras Hindu University, Varanasi 221005, Uttar Pradesh, India; Department of Microbiology, School of Life Sciences, Central University of Rajasthan, Ajmer-305817, Rajasthan, India; Enzyme Technology and Protein Bioinformatics Laboratory, School of Biotechnology, Institute of Science, Banaras Hindu University, Varanasi 221005, Uttar Pradesh, India

**Keywords:** biosurfactants, bioremediation, toxicity, emulsifying agent, eco-friendly, biodegradability

## Abstract

Globally, there is a huge demand for chemically available surfactants in many industries, irrespective of their detrimental impact on the environment. Naturally occurring green sustainable substances have been proven to be the best alternative for reducing reliance on chemical surfactants and promoting long-lasting sustainable development. The most frequently utilized green active biosurfactants, which are made by bacteria, yeast, and fungi, are discussed in this review. These biosurfactants are commonly originated from contaminated sites, the marine ecosystem, and the natural environment, and it holds great potential for environmental sustainability. In this review, we described the importance of biosurfactants for the environment, including their biodegradability, low toxicity, environmental compatibility, and stability at a wide pH range. In this review, we have also described the various techniques that have been utilized to characterize and screen the generation of microbial biosurfactants. Also, we reviewed the potential of biosurfactants and its emerging applications in the foods, cosmetics, pharmaceuticals, and agricultural industries. In addition, we also discussed the ways to overcome problems with expensive costs such as low-cost substrate media formulation, gravitational techniques, and solvent-free foam fractionation for extraction that could be employed during biosurfactant production on a larger scale.

## Teaser

In this review, we have described the various techniques that have been utilized to characterize and screen the generation of microbial biosurfactants.

## Introduction

Biosurfactants are compounds that play diverse and notable roles in different industries including soap and detergent industries, petroleum industries, and food and beverage industries and are also involved in environmental bioremediation processes (Cameotra et al. 2010). Typically, surfactants have polar heads (hydrophilic) and non-polar tails (hydrophobic), which are commonly referred to as amphipathic molecules. This property allows them to form micelles between fluids with different polarities. As an illustration, the inclusion of a surfactant causes an increase in the miscibility of oil and water, which lowers the surface tension between the two liquids (Roy 2017). The biosurfactants are surfactants that are released by microorganisms and have attracted the interest of several researchers due to their eco-friendliness and biodegradability (Shekhar et al. 2015). Chemically available surfactants have negative environmental consequences and continue to be a pollutant that causes pollution since they are difficult to bioremediate. The biosurfactants can play a vital role in augmenting the efficacy of bioremediation as they expand the surface area of substrates, form their microenvironment, and stimulate emulsification by the release of certain molecules through a variety of processes, such as quorum sensing (Gharaei-Fathabad 2011). Because of their beneficial properties, such as increased foaming potential, greater selectivity, lower toxicity and biodegradability, thermo-resistance, pH and salinity, renewable and waste-material origin, improved potency, and lack of carcinogenicity and teratogenicity, biosurfactants have proven to be far superior to oil-based surfactants (Moutinho et al. 2021). Globally, tonnes of oily waste has increased the carbon footprint and caused serious environmental issues. The oily waste is high in carbon, so using it as a base for the creation of products with added value can support the idea of carbon neutrality. Oils may be used directly as substrates and can be catabolized by microorganisms to create biosurfactants. The molecules of the twenty-first century are biodegradable and less hazardous than synthetic surfactants (Gautam et al. 2023).

Biosurfactant-producing microorganisms can have high molecular weights (proteins, lipopolysaccharides, lipoproteins, and polysaccharides) and low molecular weights (glycolipids and lipopeptides). Biosurfactants of high molecular weight have greater efficacy at stabilizing oil-water emulsions, while those of low molecular weight are good at lowering surface tension (Eras-Muñoz et al. 2022). The biological functions and uses of various biosurfactants are intimately correlated with the chemical changes in their molecular structures (Morita et al. 2015). The molecular weights of the biosurfactants produced by microorganisms range from low to high. These microorganisms include *Pseudomonas cepaci*a CCT6659 (40.5 g L−1), *Pseudomonas aeruginosa* M408 (12.6 g L−1), *Bacillus subtilis* HSO121 (47.58 g L−1), *Rhodococcus erythropolis* ATCC 4277 (0.285 g L−1), *Candida bombicola* (61 g L−1), *Candida tropicalis* UCP0996 (7.36 g L−1) (Ambaye et al. 2021). Numerous studies on *Pseudomonas sp*, a kind of bacteria recognized for producing rhamnolipids, show that many well-known biosurfactants are produced by bacteria. Because of their strong emulsification capabilities, they are often referred to as bio emulsifiers. Additionally, research confirms that fungi and yeast are also involved in the production of biosurfactants (Sáenz-Marta et al. 2015).

Biosurfactants are comprised of hydrophilic domains i.e. amphoteric or non-ionic, positively or negatively charged and hydrophobic domains which is made up of hydrocarbon chains, therefore, are amphipathic (Otzen 2017). These molecules, recognized for lowering the surface tension, produce micelles between the liquid phases of various polarities (Lombardo et al. 2015). The Most widely used surfactants are fatty acids, ethylene, ammonium salt, ethoxylate, propylene oxide copolymers, and sorbitan ester (Satpute et al. 2018). The concentration at which these surfactant domains form a supramolecular structure (micelles) calledcritical micelle concentration (CMC) and has lower CMC values than synthetic surfactants, making them more potent at low concentrations (Campos et al. 2013). In general, biosurfactants could grow progressively in harsh conditions such as pH, salinity, and temperature in industrial byproducts and waste. This capability would enable the manufacturing of biosurfactants at a reasonable cost while enabling the utilization of residual substrates to reduce environmental pollution (Carolin et al. 2021). Due to their ability to combat viruses, bacteria, and fungi, lactic acid bacteria-derived biosurfactants (LAB) have an advantage over conventional microbial surfactants. A large number of LAB strains are being linked to the production of biosurfactants, an important chemical used in the treatment of several kinds of diseases. They are also useful as anti-adhesive coating agents on healthcare insertional elements due to their efficiency as anti-adhesive agents over a wide range of bacteria, which lowers hospital infections without the usage of synthetic medications and chemicals (Thakur et al. 2023). Many researchers have been successful in isolating biosurfactants from a variety of environments, including the marine ecosystem, the natural environment, contaminated areas, and industrial wastes. Due to their unique functional characteristics, they have a wide range of uses in industries including agriculture, biomedicine, metal, construction, textiles, pulp and paper, pharmaceuticals, and cosmetics (Moutinho et al. 2021).

Recently, the demand for biosurfactants has increased manifold because of their profitable properties in different industries, including food, pharma, and agriculture. Recombinant or mutant strains with higher yields may be used as a strategy to lower substrate costs and boost productivity levels to the point where commercial production of biosurfactants eventually becomes economically viable. The use of biosurfactants as greener amphiphiles might soon be effective on a large scale (Sharma and Sharma 2021). This review article gives an insight into the technologies used in the isolation of biosurfactants and the microorganisms producing biosurfactants, as well as their significance and applications.

## Biosurfactants production and their types

Biosurfactants are mainly synthesized by microorganisms- bacteria, yeast, and filamentous fungi, but they can also be synthesized by animals (e.g. bile salts, phospholipids) and plants (e.g. saponin). Microbe-derived biosurfactants are known to have a strong emulsifying ability and lower surface tension. Because biosurfactants' composition includes biomolecules like lipids, proteins, and carbohydrates, they are more complicated structurally than synthetic surfactants (Nitschke and Pastore 2002). From an ecological perspective, biosurfactants are crucial in lowering environmental pollutants like carbon dioxide and greenhouse gas emissions. Microbes release biosurfactants during the biodegradation of hydrocarbons, which provide advantages for preserving environmental sustainability. Thereby reducing dependency on chemical degradation methods (Rahman and Gakpe 2008).

### Microbial synthesis of biosurfactant

**Figure 1.**
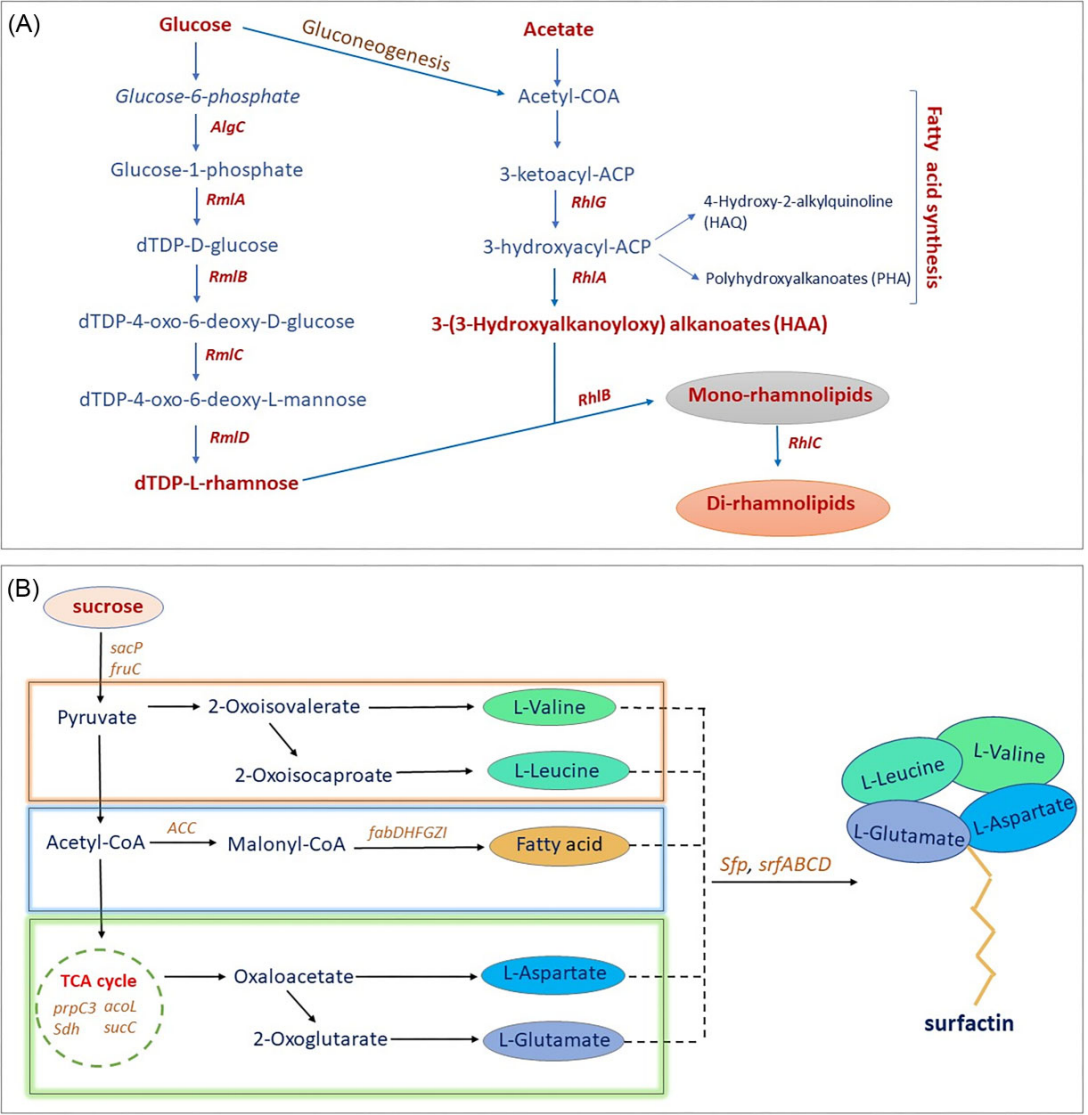
The pathway of biosurfactant synthesis. A. Rhamnolipid biosynthesis in the genus *Pseudomonas* through the metabolic process of gluconeogenesis and fatty acid synthesis. dTDP (deoxythymidine diphospahtate) L rhamnose and 3-(3-hydroxy alkonoyloxy) alkanoateare the ultimate precursor for the synthesis of mono-rhamnolipid followed by di-rhamnolipidin case of bacteria express the gene *RhlC*. B. Surfactin biosynthesis occurs in the *Bacillus* sp. using sucrose as the main carbon source which results in the production of different amino acids- L-Valine, L-Leucine, L-Glutamate, and L-Aspartate, and Fatty acid. These components are assembled into surfactin in the presence of group of genes *Sfp* and *srfABCD*in *Bacillus*.

According to reports, microorganisms employ separate paths to create the hydrophobic and hydrophilic parts of biosurfactants, which are then combined (Théatre et al. 2021). The biosynthetic pathway used by microorganisms for growth depends on the carbon source. As an example, in the case of glycolipid biosynthesis with carbohydrates serving as the only carbon source, both the lipogenic and glycolytic routes utilize carbon flow to synthesize the lipid moiety and hydrophilic part, respectively (Sánchez 2022). Many substrates are involved in the synthesis of biosurfactants (Fig. 1). Their production is influenced by changes in pH, stress, low nitrogen concentrations, and agitation rates, and can be induced by the presence of lipophilic substances. The biosurfactants rhamnolipid and surfactin, which are produced by the bacteria *Bacillus subtilis and Pseudomonas aeruginosa*, have been thoroughly researched by researchers (Sanches et al. 2021). According to several investigations, *Candida* releases lipid-derived biosurfactants made by a fungus called mannosylethitritol (MEL) (Kitamoto et al. 1993). There are different biosynthetic processes for the hydrophilic and hydrophobic domains of biosurfactants, which combine afterward (Kuo and Gardner 2002). The metabolic pathway for the production of biosurfactants mostly depends on the carbon source, which may be obtained from lipids and carbohydrates (Fig. 1). when the formation of glycolipids has utilized carbohydrates as the primary source of carbon. The energy source will then switch to the pathways for lipolysis and gluconeogenesis (Fontes et al. 2008). As an example, glycerol is used as a medium in the production of rhamnolipid, a biosurfactant produced by *Pseudomonas* (Gogoi et al. 2016). *Trichoderma reesei*, a filamentous fungus, produces hydrophobins, a surface-active globular protein, through the biosynthesis of two genes called hfb1 and hfb2. (Das et al. 2008).

The production of rhamnolipids by *P. aeruginosa* is likely the most well-known example of a bacterial glycolipid biosynthetic pathway that has been documented for both non-marine and marine strains (Tiso et al. 2017a). Two distinct glycosyltransferase units-rhamnosyltransferase I and II-catalyze the production of mono- and di-rhamnolipids. The bicistronic operon containing the genes rhlA and rhlB produces the rhamnosyltransferase I protein, although recent research has shown that both RhlA and RhlB proteins also have distinct functions (Wittgens et al. 2017). RhlB, a glycosyltransferase, catalyzes the condensation of dTDP-l-rhamnose and the 3-(3-hydroxyalkanoyloxy) alkanoic acids (HAA) to generate mono-rhamnolipids. RhlA produces HAA from activated hydroxy fatty acids. It should be noted that HAA is already surface-active metabolites that are produced from cells as biosurfactants (Tiso et al. 2017a). Rhamnosyltransferase II is encoded by the gene rhlC, which is located at a different chromosomal location in *P. aeruginosa* than rhlAB. The synthesis of di-rhamnolipid from mono-rhamnolipid and dTDP-l-rhamnose is catalyzed by this protein (Tiso et al. 2017a).

Surfactin is produced by a unique mechanism known as non-ribosomal peptide synthase (NRPS), unlike the majority of cyclic lipopeptides. The four modules that together make up NRPS are *SrfAA, SrfAB, SrfAC*, and *SrfAD*, which form a linear arrangement of seven modules. Each module is responsible for incorporating one amino acid (Youssef et al. 2005). Each module comprises of three catalytic structural domains: an adenylation structural domain (A), which selects and activates substrates; a small peptidyl carrier protein (PCP), which transfers aminoacyladenosine substrates as an enzyme-bound thioester; and a condensation structural domain (C), which forms a peptide bond within acyl-S-PCP intermediates (Ongena and Jacques 2008). Four enzyme components, *SrfA, SrfB, SrfC*, and *SrfD*, make up the surfactin synthetase complex, in which surfactin synthase is made by multi-enzymatic thio-templates and is responsible for producing surfactin. The beginning stage of surfactin production is largely controlled by *Srf D*. The surfactin synthetase operon *SrfA* is an inducible operon that promotes competence development and sporulation. The sequence of the peptide synthetase modules is consistent with the sequence of the final peptide product, and each module has a variety of domains that add or modify a particular amino acid into the growing peptide chain (Kashif et al. 2022).

### Types of biosurfactants

Biosurfactants are categorized according to their chemical composition and microbiological origin (Table 1). Mono, di, or polysaccharides, anions, or cations comprise the hydrophilic moiety of biosurfactants. In contrast, the hydrophobic moiety is made up of both saturated and unsaturated fatty acids (Abo Elsoud and Ahmed 2021). Glycolipids, the most prevalent form of biosurfactant, are composed of fatty acids and carbohydrates connected by either an ester or an ether group (Mnif et al. 2018).

**Table 1.** Types of biosurfactants and their applications

Class of biosurfactants	Subtypes of biosurfactants	Microbial origin	Applications	Reference
Glycolipids	Rhamnolipids	*Pseudomonas aeruginosa* ATCC 9027, *P. aeruginosa* GS3, *P. aeruginosa* BS2, *Bacillus sp*. AB-2, *Pseudomonas aeruginosa* FA1	Bioremediation of petroleum and heavy metals, prevent formation of biofilm, antimicrobial agent, antifungal agent, antiprotozoal, antiaging, anticancer drugs, used as a low-cost substrate made from agroprocessing waste	(Patel and Desai 1997) (Herzog et al. 2020) (Kareem and Mussa 2020) (Kashif et al. 2022) (Zhao et al. 2023)
	Sophorolipids	*Torulopsisbombicola* ATCC 22214, *T. apicola* and *T. petrophilum* *Candida* sp. SY 16, *Eucalyptus camaldulensis*	Antifungal activity, biocontrol agent, cosmetics, increase shelf life of bread, antialgal, antimycoplasma, antifungal agent and desorption of polycyclic aromatic hydrocarbons (PAHs).	(Kim et al. 2006) (Li et al. 2020) (Kareem and Mussa 2020) (Kariyawasam et al. 2023)
	Trehalolipids	*Nocardia, Mycobaterium* and *Corynebacterium, Rhodococcuserythropolis* DSM 43215, *R. erythropolis* 3C-9*, Rhodococcus sp*. PML026, *Rhodococcusruber* IEGM 231*, Fusariumfujikuroi* UFSM-BAS-01.	Antitumor effectiveness, inducting the murine macrophages to outputted nitric oxide (NO-), exhortation the cytokines productions, consolidation the antigenic activity and participate in the regulation of T-cell immunity.	(Peng et al. 2007) (Reis et al. 2018) (Kuyukina and Ivshina 2019) (Kareem and Mussa 2020) (Jimoh et al. 2021) (Gein et al. 2022)
Lipopeptides	Surfactin	*Bacillussubtilis* A21, *Bacillus pumilus* A1, *Bacillus megaterium* ATTC 14581	Anti-tumor activity against the human breast cancer cells, anti-endotoxin agent, surfactin for oral delivery of insulin, implicated in the bioaccumulation and biosorption of Pb and Ar.	(Morikawa et al. 1992) (Hwang et al. 2007) (Duarte et al. 2014) (Zhang et al. 2016) (Fei et al. 2020) (El-Sheshtawy et al. 2022)
	Arthrofactin	*Actinomyces, Arthrobacter* and *Streptomyces*.	Antimicrobial action by disrupting microbial biofilm.	(Sari et al. 2020)
Polymeric biosurfactants	Emulsan	*Acinetobacter* *Calcoaceticus, Acinetobacter* *Calcoaceticus* BD4, *Acinetobacter calcoaceticus* PTCC1318	Microbially enhanced oil recovery (MEOR), treatment of dental plaque, phosphate removal from waste-water.	(Kaplan et al. 1987) (Gutnick et al. 1991) (Amani and Kariminezhad 2016)
Fatty acids and phospholipids biosurfactants	Phospholipids	*Corynebacterium Lepus*	Cosmetics, household formulations and bioremediation.	(Saharan et al. 2011) (Busi and Rajkumari 2017) (Lee et al. 2018)

### Glycolipids

One of the most common types of biosurfactant is glycolipid. They originate from lipids and are composed of lengthy chains of hydroxyaliphatic or aliphatic acid-containing sugars. Well-known subtypes of glycolipids include rhamnolipids, sophorolipids, and trehalolipids (Roy 2017). *Pseudomonas aeruginosa* is primarily responsible for producing rhamnolipids, which have a wide range of uses in bioremediation (Costa et al. 2010). It consists of one or two rhamnose sugar groups resulting in the formation of mono- or di-rhamnolipid molecules (Raza et al. 2010). Another subclass of glycolipids that function as biosurfactants on extracellular surfaces is called sophorolipids. The two carbohydrate sophorose units that makeup sophorolipids are joined to long-chain fatty acids via a glycosidic bond. It has been discovered to be quite helpful in oil bioremediation procedures(Elshafie et al. 2015). Although sophorolipids can lower interfacial and surface tension, they are ineffective emulsifiers (SajadiBami et al. 2022). The most prevalent sophorolipids are those made by yeast such *Torulopsis bombicola, T. apicola*, and *T. petrophilum* (Shekhar et al. 2015). Trehalose units (a disaccharide unit), which are discovered to be connected to mycolic acids, make up the glycolipids known as trehalolipids. The size, mycolic acid structure, level of unsaturation, and number of carbon atoms of trehalolipids might vary depending on the organism that produced them (Banat et al. 2010). There are several species, including *Nocardia, Mycobacterium*, and *Corynebacterium*, that are involved in the production of trehalolipids (Kuyukina and Ivshina 2019).

### Lipopeptides

Lipopeptides are surface-active biosurfactants with antimicrobial properties (Khan et al. 2019). The most effective cyclic lipopeptides are surfactin and arthrofactin (Nakamoto et al. 2021). Surfactinis is mostly produced by the bacteria *Bacillus subtilis* and has a lengthy carbon chain with less hazardous amino acids(Fei et al. 2020). Due to its antibacterial, antiviral, and antimicrobial qualities. It has been employed in a variety of sectors, such as the cosmetic and oil bioremediation processes(Drakontis and Amin 2020). Arthrofactin is also a type of lipopeptide biosurfactant produced by *Actinomyces, Arthrobacter*, and *Streptomyces* (Sari et al. 2020). It is effective at preventing the growth of biofilms by reducing the surface tension of water from 72 to 24 mNm-1 (Lange et al. 2012).

### Polymeric biosurfactants

Polymeric biosurfactants such as extracellular polymeric substances (e.g. emulsan) can have deleterious and productive effects during microbial biofilm and floc formation (Sajadi Bami et al. 2022). They are known to alter surface characteristics during biofouling, including hydrophobicity, color, roughness, and frictional resistance. In the process of biocorrosion of metals, the EPSs can bind with the metal (Vimalnath and Subramanian 2018).

### Fatty acids and phospholipids biosurfactants

When many bacterial and yeast species are grown on n-alkanes or hydrocarbon substrates, they produce huge amounts of fatty acids and phospholipids (Sajadi Bami et al. 2022). Several lipids are produced by them, such as lipopeptides, glycolipids, and phospholipids (Hausmann and Syldatk 2014). *Corynebacterium Lepus*is responsible for the production of the most popular phospholipid biosurfactants (Busi and Rajkumari 2017).

## Applications of biosurfactants

Because they are effective wetting and foaming agents, solubilizers, dispersants, and emulsifiers, as well as detergents, biosurfactants' qualities may be used widely on the commercial scale (Banat et al. 2010). The market's availability of chemicals-based products has decreased as the demand for bio-based products has increased (Olasanmi and Thring 2018). This has been determined by the expansion of bio-based chemical patent rights (Tiso et al. 2017).

### Industrial uses of biosurfactants

Biosurfactants are used in several industrial processes, including biorefinery and cooling. Ice slurry is a homogeneous substance made up of water and tiny pieces of ice. Processes including cooling, air conditioning, and cold storage systems are all necessary for the development of ice slurries. By stabilizing them in an ice-water slurry, di-acetylated MELs inhibit the build-up of tiny ice particles. MELs are added to biodiesel to improve its flow characteristics, which improves its performance at low temperatures (Madihalli et al. 2016). Processes in biorefineries utilize biosurfactants because they potentially speed up the biodegradation of complicated biomass. Their presence may also hasten the pace at which cellulases break down lignocellulose as a result of their strong binding (Liu et al. 2017).

In microbial enhanced oil recovery (MEOR), secondary metabolites of microbial originsuch as acids, enzymes, solvents, gases, biopolymers, and the most assuring biosurfactants are used to substitute synthetic surfactants in recovering secondary oil from sedimentary rock (Sarubbo et al. 2022). Ex-situ and in-situ biosurfactant production are effective approaches to MEOR. In-situ biosurfactant production typically begins with the injection of microorganisms which produce biosurfactants, followed by the infusion of nutrients into the reservoir. Industrial bioreactors can also be used to produce ex-situ biosurfactants for subsequent infusion of these substances into the reservoir using CO_2_. Microorganisms produce emulsifiers and surfactants that reduce surface tension and cause the trapped oil to escape. By improving the flushing efficiency of the infiltrated fluid and CO_2_, biosurfactants change the water-holding capacity of the CO_2_ that's been injected and the behavior of CO_2_-brine-rock at the interface, which facilitates the recovery of the oil (Selva Filho et al. 2023). In the course of MEOR, nutrients are added to the oil reservoir together with microorganisms capable of producing biosurfactants to promote microbial development (Sun et al. 2018). Biosurfactanat are significant at mobilizing immobile hydrocarbons as it can decrease the surface tension across the oil and rock, lowering the capillary forces to transport of oil throughout rock pores (Rawat et al. 2020). Rhamnolipid extracted from *P. aeruginosa* helped to recover medium-weight oils at a rate of 50.45%, an improvement of 11.91% made possible by the presence of the microbe. Here, this microbial application has shown higher recovery rate than than that attained with the tested synthetic surfactants (Câmara et al. 2019). Sometimes, the use of biosurfactants in MEOR is a contentious matter because the biosurfactants required to remove oil residues that are trapped in the porous rocks, are undue and it may not be cost effective. Yet, it is considered counterintuitive to use substances for oil recovery whose primary benefit is to take the place of synthetic chemicals in the petrochemical industry (De Almeida et al. 2016).

There are numerous studies conducted on the use of biosurfactant for the cleanup of oil-contaminated soil. Surfactin is one of the surfactants employed in biotechnological methods of decontamination, with 85% removal by utilizing *Bacillus licheniformis* derived biosurfactant and 88% with biosurfactant derived from *Bacillus subtilis* (Alvarez et al. 2015, Khademolhosseini et al. 2019). In bioremediation, biosurfactants must undergo biodegradation in soils, which makes them a good ecological replacement for synthetic surfactants. Biosurfactants are adept at technology and can be discharged in-situ, where they may carry out their effects with less subsequent handling effort than their synthetic surfactants (Silva et al. 2020).

### Medical applications

Lipopeptides are biosurfactants known for their stability over a wide pH range and heating them at high temperature does not result in the loss of their surface-active property. Recent studies show that they also possess antimicrobial properties. For example, rhamnolipid which are produced by the genus *Pseudomonas* are known to have powerful antimicrobial properties as mono-rhamnolipids have a bacteriostatic effect, di-rhamnolipids exhibit a bactericidal effect on *P. aeruginosa* (Diaz et al. 2016). There are some biosurfactants which are having a synergic effect on antibiotics by augmenting their uptake efficiency into the cell (Hage-Hulsmann et al. 2018).

Due to their antibacterial, anti-adhesive, and enzyme-inhibiting qualities, biosurfactants have been employed in the medical and pharmaceutical sectors for a variety of therapeutic purposes (Markande et al. 2021). Gene-releasing biosurfactants, pharmaceuticals, and also antiviral properties, and anticancer activities are some of the key areas of study involving these biomolecules in the fields of pharmacy and medicine (Drakontis and Amin 2020). Recent research has focused on COVID-19 management tactics and investigated the use of biosurfactants as cleansers, disinfectants, environmentally friendly sanitizers, antiviral agents, and anti-inflammatories. Sophorolipids may be used as therapeutic agents to combat the SARS-CoV-2 virus, according to a new study (Daverey et al. 2021). SARS-CoV-2 is a positive sense single stranded RNA envelope virus which can be pleomorphic or spherical. The four main structural proteins (NC-nucleocapsid protein, M-membrane protein, E-encapsulation protein, and S-spike protein) as well as five to eight nonstructural auxiliary proteins are all encoded by the genome. The ACE2 receptor location on the epithelial cells that line the respiratory tract of the host is where the spike glycoprotein attaches to the viral particle (Baglivo et al. 2020). The anionic character of sophorolipids disturbs the viral envelope, causing the structural elements to disintegrate and consequently interfering with the connections between virus protein surfaces and cell-host receptor sites (Kashif et al. 2022). Additionally, they are found to be suitable for usage in future innovations like nanobiotechnology and effective medication delivery systems. Also, they have drawn considerable interest from the scientific community due to their strong therapeutic qualities, making them useful in the treatment of SARS-CoV infection as well as for anti-viral, immunomodulatory, anti-cancer, wound healing, and other conditions (Kumari et al. 2023).

Biosurfactants are often employed in antimicrobial and anti-viral research and also in drug delivery. The use of biosurfactants as potential substitutes to manage biofilms has been thoroughly investigated in recent years. Biosurfactants change the surface characteristics of bacterial cells that are connected with their membranes and prevent them from adhering to other substrates. Additionally, it has been shown that biosurfactants generated by Gram-negative bacteria prevent microorganisms from developing biofilms and communicating with one another (Kashif et al. 2022). The biosurfactants have antibacterial action against a variety of pathogenic pathogens, including *Candida albicans, E. coli*, and others. According to a new study conducted by Gupta et al., a glycolipid biosurfactant made from *B. licheniformis* SV1 showed fast wound-healing action (Gupta et al. 2017). Similarly, another study found that the administration of lipopeptide biosurfactants from *Acinetobacter junii* B6 improved the rate of wound healing in rats (Ohadi et al. 2017). The antioxidant characteristics of the biosurfactants, which lessen oxidative stress by reducing the production of reactive oxygen species and increasing the activities of free radical scavengers, may be the cause of the rapid wound healing when provided with lipopeptides. Because the structure includes both a fatty acid chain and a peptide group, lipopeptide biosurfactants also have anticancer action. Due to their potential involvement in apoptosis, cell proliferation, signal suppression, and cell cycle inhibition, biosurfactants have lately been examined for their anticancer characteristics (Kashif et al. 2022).

### Cosmetic applications of bio-surfactants

Biosurfactants have vital physiochemical properties for preserving healthy skin. For instance, their molecules' fatty acid ends help moisturize the skin's rough and dehydrated surfaces. The accessible fatty acids may also function as anti-oxidants, which would prevent the creation of free radicals brought on by UV radiation (Thakur et al. 2021). Skin infection pathogens including *Staphylococcus aureus, P. aeruginosa, Candida acnes*, and *Streptococcus pyogenes* have all been demonstrated to be effectively suppressed by a variety of biosurfactants. Because of this, biosurfactants are being suggested as a potential substitute for conventional antibiotics, even though their bactericidal activity can often be weak (Loeto et al. 2021). Their usage in cosmetic industries is major because of their wetting, foaming solubilizing, dispersing, and emulsification properties (Varvaresou and Iakovou 2015). The biosurfactants are highly compatible with the skin and also don't cause any irritation. The antimicrobial properties of biosurfactants proved to be a boon for cosmetic products (Lourith and Kanlayavattanakul 2009). It has been discovered that the carbohydrates, lipids, and proteins included in biosurfactants are similar to the membrane found on skin cells (proteins and phospholipids). Due to their unique structural property, biosurfactants can cross the skin cell membrane, which can activate potential benefits on hair repair, skin protection processes, and the regulation of protein skin barrier functions. The movement of substances across the skin cell membrane is controlled by lipophilicity and interfacial properties (Ferreira et al. 2017).

Large cosmetic corporations often provide 10 000 distinct cosmetic items, and 25–30% of these goods are reformulated annually. The use of novel active components for consumers or the industry is a factor in around 10% of these reformulations. These businesses add up to 80 new components to their product line per year (Sarubbo et al. 2022). In the aforementioned context, using biosurfactants is one way to satisfy the need for novel components. RelipidiumTM (a body and face moisturizer made by BASF in Monheim, Germany), SopholianceTMS (a deodorant, face cleanser, and shower gel made by Givaudan Active Beauty in Paris, France) and Kanebo skincare, (a moisturizer, cleanser, and UV filter made by Kanebo Cosmetics in Tokyo, Japan) are a few products which incorporate these biomolecules (Adu et al. 2020). Additionally, Evonik, a German chemical company is now set technologies for the production of rhamnolipid as foam promoters in cosmetic products, assurance the applicability of biosurfactant as one of the safety and active ingredients in formulation of cosmetic products. In 2010, Evonik was able to develop biotechnological techniques for producing microbial biosurfactants on a commercial level (Sarubbo et al. 2022). In the current circumstance, using biosurfactants is one way to satisfy the need for novel components as they represent minimal dangers to humans and the environment due to their biodegradable, renewable, or non-toxic nature, which is in the interests of the developing consumer segment and, as a result, the cosmetic business. Investment in the practical study of such biomolecules has a good possibility of producing reformulations and the creation of novel, safer cosmetics (Olasanmi and Thring 2018). Glycolipid and lipopeptide biosurfactants more broadly have a low level of toxicity, and antimicrobial, and dermatological moisturizing properties that make them a great deal better than chemical surfactants in recent demand for cosmetic and personal skincare products (Adu et al. 2020).

Kao Co. Ltd. in Japan is a prominent producer of sophoolipid substances for use as a humidifying agent in different commercial goods such as hair moisturizers, skin moisturizers, and lipsticks. Sophorolipids can encourage hair regrowth and preserve the skin (Adu et al. 2023). These biosufactant may also reduce fat deposits in the skin by boosting adipocytes' production of the hormone leptin. According to various research, rhamnolipids are also thought to be biocompatible and a prospective component for usage in medical formulations of cosmetics as well as personal dermatological products (Karnwal et al. 2023). Human keratinocyte cells, which are crucial for skincare applications, were studied by Adu et al. to compare the effects of manufactured surfactants (sodium lauryl ether sulphate) and naturally produced glycolipid biosurfactants (rhamnolipids and sophorolipids). The results show that the acidic nature of mono-rhamnolipids and sophorolipids very modestly affects cell survival and inflammatory cytokine production, but the impact of various glycolipids on cells varies depending on their chemical makeup. Di-rhamnolipids have been shown to effectively minimize inflammation and increase the anti-inflammatory action of cytokines at noninhibitory concentrations, making them a potential replacement for chemical surfactants in skin care products and helpful for dermatological conditions like psoriasis (Adu et al. 2023). According to Etemadzadeh et al. (2023), salt-tolerant *Bacillus halotolerans* produces a biosurfactant that shows several therapeutically relevant properties and may be used as a raw ingredient in the manufacture of food, pharmaceutical, and cosmetic products. In vitro studies on the derived lipopeptide showed that 90.38% effectiveness at 0.8 mg/mL with antibacterial and antioxidant properties. Besides, it has anticancer potential through induction of apoptosis in MCF-7 cells while possing no negative effect on unharmed HEK-293 cells (Etemadzadeh et al.2023).

### Food industries

Biosurfactants play a very important role in food industries because of their non-toxic nature and easy biodegradability. To feed the growing population, it is necessary to increase the productivity of agriculture. They also contribute further to the production of food, which includes (i) soil improvements, (ii) stimulation of effective foliar fertilizers uptake, (iii) protection from phytopathogens, (iv) amelioration microbe-plant interactions (Sachdev and Cameotra 2013, Liu et al. 2016). They have several useful applications in the manufacturing of food and also possess antibacterial and anti-biofilm properties that are utilized for sanitization and to prevent food from spoiling (Kiran et al. 2017). Silva et al. used biosurfactants to substitute 50%–100% of the plant fat in cupcakes as part of a bakery-related application. Through the removal of trans-fatty acids, the nutritional content of the cupcake was somewhat improved by the substitution of plant fat with a biosurfactant (Silva et al. 2020). The main reasons why food is wasted each year throughout the world are the deterioration in food quality, microbiological contamination spoilage, and the short shelf life of particular goods. The usage of biodegradable packaging and antimicrobial additives are only a couple of the many strategies being investigated right now to deal with these problems (Cofelice et al. 2019). Kourmentza et al. recently reported the antibacterial potential of the lipopeptide BS against foodborne pathogens such as *Bacillus sp*. Additionally, they reported antibacterial activity against the filamentous fungus *Byssochlamys fulva, Candida krusei*, and *Paecilomyces variotti* (Kourmentza et al. 2021). The yeasts *Starmerella bombicola, Meyerozyma guilliermondii Candida sphaerica*, and *Saccharomyces cerevisiae* are among the microorganisms that have recently been reported for the production of biosurfactants. These organisms have the potential to produce substances with emulsifying properties and surfactant activities along with antibacterial and antioxidant qualities (Ribeiro et al. 2020). Regardless of the wide range of food application possibilities, various studies are necessary to develop a practical application that can execute functions in complicated food matrices under diverse processing circumstances. It's crucial to create methods that use these biomolecules at the most modest feasible concentration for optimum performance for an affordable application (Augusto et al. 2020).

### Bioremediation

**Figure 2.**
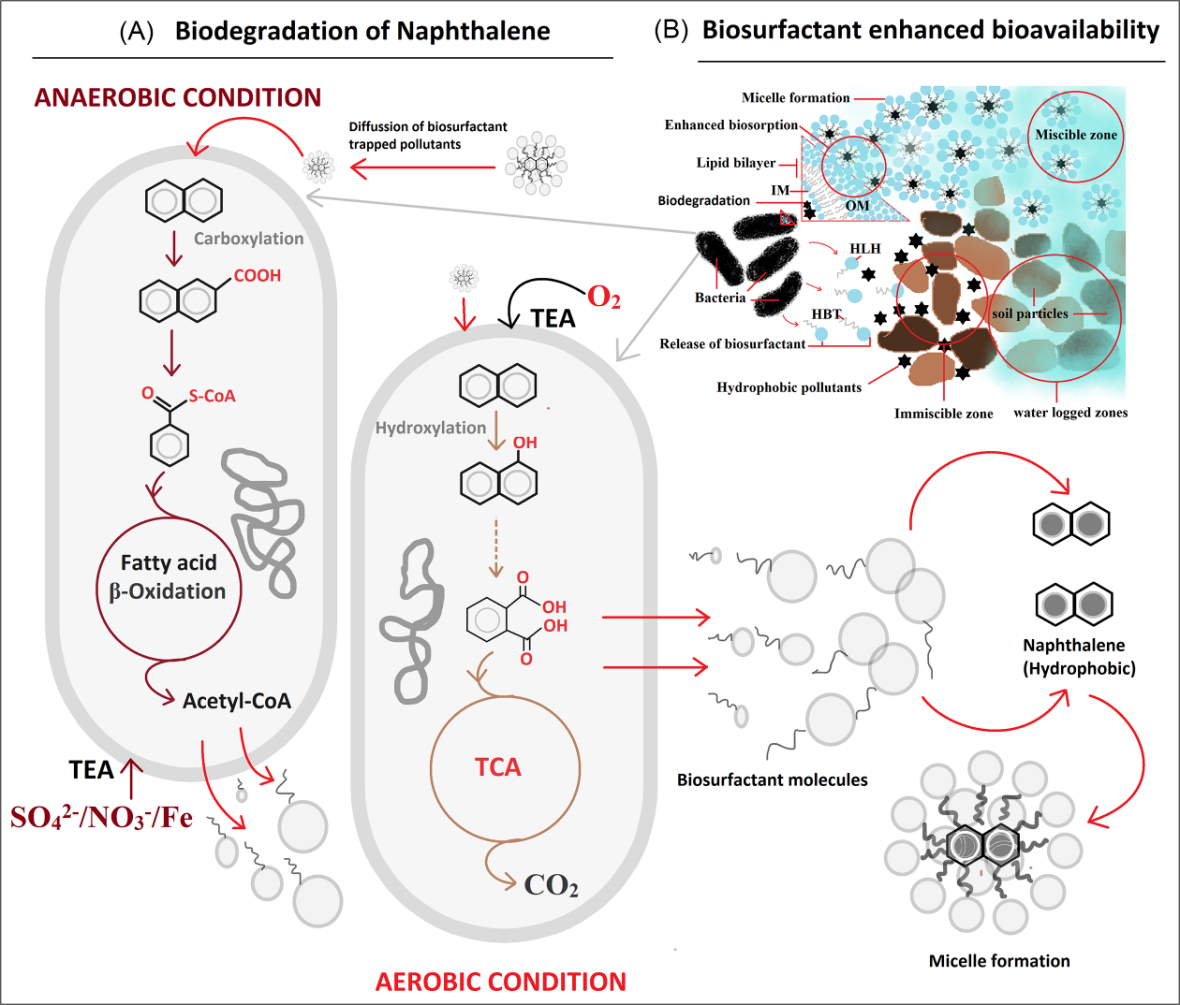
Enhanced bioavailability and biodegradation of hydrophobic pollutants through release of biosurfactant. IM (Inner Membrane), OM (Outer Membrane), HLH (Hyrophilic Head), HBT (Hydrophobic Tail).

Biosurfactants are capable of withstanding extremes in temperature, salinity, and environmental abrasion while maintaining their stability. Petroleum and heavy metals are detoxified from the contaminated environment utilising biosurfactant-based remediation approaches and microorganisms that produce biosurfactants (SajadiBami et al. 2022). Also aids in the dissolution of hydrophobic pollutants in water. They have been shown to be the best substitute for chemically manufactured surfactants used in bioremediation because of their inherent low toxicity and great biodegradability (Mao et al. 2015). The emulsification property and higher solubility of biosurfactants promote cellular utilizations of contaminants (Shah et al. 2016). Organic and metallic pollutants are unavailable to microorganisms for breakdown, which is one of the reasons why they persist in soil for such a long period without being eliminated. Additionally, the pollutants' interactions with the environment and the microbe may be insufficient, which prevents the microorganism from carrying out the required catabolic processes. Therefore, biosurfactants produced by bacteria and fungi play a crucial role in solubilizing hydrophobic contaminants, allowing for their direct elimination (Abbot et al. 2022). The biosurfactants produced by *Stenotrophomonas* sp. S1VKR-26 can be utilized to bioremediate wastewater contaminated with petrolatum (Patel and Patel 2020). The lipopeptide-type biosurfactant *Bacillus cereus* UCP 1615 can clean up oil spills (Durval et al. 2020). The hydrophilic compound's solubility is increased by the biosurfactant that was isolated from *Rhodococcus erythropolis* HX-2, which also speeds up the biodegradation of petroleum (Hu et al. 2020). The release of biosurfactant in the soil helps to enhance the biodegradation process in bacteria, as schematically represented in Fig. 2.

## Significance of biosurfactants

Microbes are essential in the production of biosurfactants. Due to their microbial origin, they offer several beneficial characteristics, including low toxicity, high stability, eco-friendliness, simple biodegradability, the ability to function at severe temperatures, and the capacity to withstand a wide range of pH.

### Environmental compatibility

Environmental pollution is a major concern nowadays because of the increasing population. To control pollution, we have to spread awareness among people that will contribute towards cleaning up environmental pollution. About two decades ago, it was found that there are applications of biosurfactants that can be used for environmental sustainability. Also play a very crucial role in the removal of major greenhouse gas i.e. carbon dioxide from the atmosphere (Rahman and Gakpe 2008). Chemically produced surfactants found to deplete non-renewable petrochemical resources (Henkel et al. 2012). Accumulation of their counterparts puts the environment in danger because they are non-biodegradable (Rahman and Gakpe 2008). Hydrophobic contaminants produced by the oil and gas sector can collect in soil. Soil contamination can be caused by a variety of things, including storage tank leaks, spills, and pipeline leaks from accidents involving oil exploration, refinement, and shipment. In addition to being poisonous, these contaminants are also obstinate, intractable, and hazardous (da Silva Faccioli et al. 2022). Surfactants enable contaminants to desorb from soil particles and favor their mineralization and microbial breakdown by lowering their surface and interfacial tensions. Another method of contamination removal is phytoremediation, which involves plants absorbing contaminants with the aid of biosurfactants (Fenibo et al. 2019). The *P. cepacia* CCT6659 biosurfactant showed potential for use in the biological remediation of soils. In trials with soil polluted with hydrophobic organic matter, an indigenous consortium and biosurfactant treatment resulted in the breakdown of 95% of the contaminants within 35 to 60 days (Silva et al. 2014a). Additionally, biosurfactants are frequently used to clean the soil, increase the amount of nutrients in the soil, and function as biocides, focusing on bacteria. Pesticides are made more bioavailable by biosurfactants, which speeds up the process by which these sediment and soil contaminants degrade (Rawat et al. 2020). They are also involved in the remediation of heavy metals. Heavy metal removal rates were achieved with the use of biosurfactants (41% for Ni, 30% for Cr, 29% for Pb, and 20% for Zn), and removal rates of Fe, Zn, and Pb from soil using an anionic biosurfactant made by *C. sphaerica* UCP0995 in various combinations with NaOH and HCl were 95, 90, and 79%, respectively (Luna et al. 2016, Liduino et al. 2018). According to many reports, using biosurfactants in these formulations is a sustainable substitute for synthetic surfactants. *P. aeruginosa* rhamnolipids can be utilized to stabilize water-in-diesel emulsions to be utilized as fuel. This mixture is required to lessen viscosity during the transportation of diesel, the primary energy source, as well as to lessen emissions of fine particulates and gases containing hydrocarbons (Fenibo et al. 2019).

### Low toxicity

Synthetic surfactants after use are released into wastewater streams which imposes a threat to the ecosystem (Ivanković and Hrenović 2010). The damage caused by synthetic surfactants to the water body depends on their concentrations. As increased concentration leads to the growth of algae by which other microorganisms' cell membranes become more permeable and result in its disintegration (Yuan et al. 2014). Glycolipid and lipopeptide biosurfactants broadly have low toxicity, antimicrobial, and skin surface moisturizing properties that portrayed as suitable substance in replace of chemical surfactants in cosmetics and skincare products (Adu et al. 2020). The toxicity imposed by surfactants depends on their hydrophobicity. Due to the increase in toxicity levels, there is a high chance that it will be consumed by animals and disturb the animal food chain. Aquatic animals such as fishes absorb these surfactants through their body surface and gills and then pass it to the blood circulation. When consumed by humans through food, it will lead to damage to enzyme activity (Ivanković and Hrenović 2010). Analyzing the usage of surfactin produced from *Bacillus subtilis* HSO121 indicated that this compound is a non-toxic, non-irritating substance, making it a safer one to be employed in detergent formulations (Fei et al. 2020). A comparison of Marlon A-350, a synthetic surfactant, and a biosurfactant produced by *Pseudomonas aeruginosa* revealed that the biosurfactant was non-toxic while the synthetic surfactant was extremely harmful in all experiments (Muthusamy et al. 2008). Santos et al. discovered that biosurfactant samples obtained at 0.02 and 0.06% did not significantly increase the fatal rates for *Artemia salina*, whereas 0.08% resulted in the death of 100% of all the larvae (Santos et al. 2017). In a study by Santos et al. biosurfactants produced by *Streptomyces sp*. DPUA1559 in concentrations of 50, 100, and 150 mg/mL with CMC (10 mg/mL) was shown to have no detectable fatal rates (Santos 2013). Because of this reason, biosurfactant discovery proved to be a boon against synthetic surfactants.

### Biodegradability

Biosurfactants derived from microbes can easily undergo the process of bioremediation or biosorption in comparison to synthetically available surfactants (Desai and Banat 1997). Marine microorganisms producing biosurfactants are best for biosorption of solvent polycyclic sweet-smelling hydrocarbons and contamination caused by phenanthrene over aquatic surfaces (Gharaei-Fathabad 2011). Microbial surfactants are susceptible to biodegradation because of their natural origin and do not accumulate in soil and water. The enzymatic activities of certain microorganisms break down these surface-active substances by first cleaving and then inactivating the surfactant monomers. Surfactant monomers are known to be broken down by a number of enzymes (Kashif et al. 2022). The polysaccharide backbone of emulsions, for instance, is broken by the enzyme emulsan polymerase, rendering the molecule inactive (Santos et al. 2016). According to research that looked at the biodegradability of sophorolipids made by a non-pathogenic strain of *Candida bombicola*, biosurfactants degraded instantly in comparison to synthetic surfactants, which continued to function even after eight days (Ahn et al. 2016). Rhamnolipids were discovered to disintegrate in both aerobic and anaerobic environments; whereas, the synthetic surfactant (Triton X-100) only decayed partially in aerobic environments and failed to degrade anaerobically (Kashif et al. 2022). There is a study conducted by Moldes et al. for bioremediation of soil contaminated with octane. The biosurfactants produced by *Lactobacillus pentosus* results in a reduction of octane concentration in soil nearly to 60% after 15 days of treatment. The biodegradation rate of octane increased to 76% after 30 days of treatment. While the removal rate of octane is three times slower in the absence of biosurfactants (Moldes et al. 2011).

### Temperature and pH tolerance

Biosurfactants produced by microbes that live in extreme conditions can tolerate a wide temperature and pH range. Surface tension can be readily reduced by biosurfactants at higher temperatures, but it is more difficult to do so in low-temperature conditions. To overcome the temperature barrier and reduce surface tension, it is, therefore, reasonable to deduce that microorganisms from cold environments will have a longer, less-branched hydrophobic chain (Antonioli Júnior et al. 2022). Depending on the environment's temperature, pressure, pH, structure, and level of solution stability, lipopeptides' emulsification capabilities might vary (Kumar and Ngueagni 2021). For instance, pH can affect the emulsifying action of surfactin formed by *B. subtilis*. It produces an emulsion that remains stable with kerosene at pH levels over 7, however, if the pH falls below 3, the emulsion is not formed (Long et al. 2017). The isolated *Planococcus sp*. XW-1 showed exceptional ability in the production of surfactants at low temperatures and the degradation of petroleum. Introducing *Planococcus sp*. XW-1 at 4°C caused the degradation of 54% of crude oil. These results indicate that *Planococcus sp*. XW-1 is a good option for in-situ bioremediation of marine ecosystems contaminated with petroleum in the north Yellow Sea during the winter (Guo et al. 2022). A study conducted on lichenysin produced by *Bacillus licheniformis* found it to be resistant up to a temperature of 50°C and having a pH range of 4.5 to 9 and can tolerate NaCl and Ca concentrations up to 25 to 50 gL^−1^ (Purwasena et al. 2019). The biosurfactant produced by *Bacillus subtilis* strain JA-1 is found to have surface activity and emulsification capability that are stable even at pH levels of 7–8. *Pseudomonas aeruginosa* RS29 developed a biosurfactant that demonstrated pH, saline and temperature stability. Even under these harsh circumstances, it was said to have significant foaming and emulsifying capabilities (Rufino et al. 2008). Since industrial applications require a wide pH and temperature range for their procedures, it becomes more important to focus on novel microbes that will produce under extreme conditions (Das and Mukherjee 2007).

## Techniques used for screening of biosurfactants

Biosurfactants can be isolated from different locations such as oil fields, petroleum and hydrocarbon-contaminated sites, garbage soil, thermophilic and halophilic environments, marine environments, and many more other sites yet to be explored. As for example, *Pseudomonas aeruginosa* SP4 has been isolated from petroleum-contaminated soil (Pornsunthorntawee et al. 2008), *Pseudomonas* and *Bacillus* sp found to be isolated from soil contaminated with domestic wastewater (Femi-Ola et al. 2015), *Vibrio sp. LQ2* was isolated from cold-seep sediment (Zhou et al. 2021) and *Azotobacter chroococcum* was isolated from the marine environment (Thavasi et al. 2009).

Screening of biosurfactants involves many methods (Table 2). Morais et al. performed the drop collapse method to observe drop collapse activity of crude oil shown by culture supernatant using glass slide (Morais et al. 2017). The oil displacement technique reported by Satpute involved adding 2 mL of crude oil and 50 mL of distilled water to a Petri dish in such a way that the oil is distributed evenly across the water's surface. Afterwards, a culture supernatant of 500 µL was added and the presence of biosurfactant shows clear zones on the oil surface (Satpute et al. 2018). Lipase assay was performed by Kumar and his colleagues with modifications,10 µL of overnight culture broth was added to tributyrin agar medium plates, which were then incubated at 37°C for 48 hours. Around colonies producing biosurfactants, the zone of lysis was seen (Kumar et al. 2017). The penetration method can be used as a primary screening method for biosurfactants, 96 well ELISA microplates were taken. A hydrophobic paste made with oil- 200 µL and silica gel was poured into each well, the activity of biosrurfactant was observed by the addition of crude oil (10 µL), of culture supernatant (90 µL), and 10 µL of safranin solution (Kumar et al. 2017). The hemolytic activity method proved to be a clear indicative test for biosurfactant production by many authors (Tabatabaee et al. 2005). This procedure involved incubating culture broth for an overnight period, inoculating 10 L of culture over 5% sheep blood agar, and then incubating the plates at 37°C for 48 h. A lysis zone was seen surrounding the colonies (Roy 2017). The presence of biosurfactants can be measured by emulsification index (EI%) and emulsification assay. To calculate the emulsification index (EI%), a 48-h-grown culture was centrifuged at 10 000 rpm for 10 min to collect supernatant. The equal amount of culture supernatant and crude oil are mixed and vortexed for 15 minutes at room temperature, allowed to stand for 24 h at dark chamber and determined the emulsification index (Dusane et al. 2011). In the emulsification assay, 3 mL of culture supernatant and 0.5 mL of oil were placed in a test tube, vortexed for 5 min, and then left at room temperature for 1 h. After that, the mixture's aqueous phase was collected, and an absorbance measurement at 400 nm was taken (Campos et al. 2015). A DuNouy tensiometer was used to measure surface tension using the ring technique at room temperature. The critical micelle concentration (CMC) method was utilized to determine the surface tension property of biosurfactants. A certain concentration of biosurfactants known as CMC results in the formation of micelles. Different concentrations of the extracted biosurfactant (0–500 mg/L) were created with the use of distilled water, and the surface tension was measured (Sambanthamoorthy et al. 2014). In the oil-coated agar plate method, oil coating was applied over the surface of the nutrient agar media plate. Followed by streaking of a given isolated strain and leaving the plates for incubation for 7 days at 37°C. The presence of an emulsification halo around the culture growth plate shows the activity of biosurfactants (Burd and Ward 1996). The chemical composition and component analysis of the isolated biosurfactant is done by FTIR spectroscopic analysis (Shimadzu, Japan). Using this technique, samples were created by mixing potassium bromide pellets with 1 mg of biosurfactant in a homogeneous suspension. The infrared spectrum was determined by using an integrated plotter. With a resolution of 4 cm^-1^, the IR spectrum's range was 450–4500 cm^-1^ (Ferreira et al. 2017).

**Table 2.** Methods for screening of biosurfactant and its significance.

Methods	Protocol	Significance	References
Drop collapse method	Drop collapse activity was observed by adding a drop of crude oil and culture supernatant onto the glass slide	A straight forward and practical approach that provides a delicate yet quick manner of producing biosurfactant and evaluation of the presence of biosurfactants can be done on a qualitative and quantitative level0	(Jain et al. 1991) (Plaza et al. 2006) (Morais et al. 2017)
Oil displacement method	50 mL of distilled water was poured into the Petri dish and 2 mL of crude oil was added in such a way it distributes uniformly on water surface. Afterwards, culture supernatant of 500 µL was added and biosurfactant activity can be seen by clear zone on the oil surface	Most widely used technique for quick and simple preliminary detection of bacteria that produce biosurfactants and it can be used when the biosurfactant activity and quantity are limited	(Plaza et al. 2006) (Walter et al. 2010) (Satpute et al. 2018)
Hemolytic activity	Culture broth were incubated overnight, 10 µL of culture inoculated onto 5% sheep blood agar and then incubated the plates at 37°C for 48 h. The zone of lysis was observed around the colonies	Used as a primarily screen test for biosurfactant production. However, it is not a very accurate approach for biosurfactant production test in bacteria since many good biosurfactant producersare left out due to negative haemolytic zone on blood agar and also non biosurfactant producers also showed hemolytic activity.	(Thavasi and Jayalakshmi 2003) (Roy 2017)
Emulsification index (EI%)	48 h grown culture was subjected to centrifugation at 10,000 rpm for 10 min. After centrifugation, 2 mL of culture supernatant were taken and followed by addition of 2 mL of crude oil to it. Then whole assembly was vortexed for 15 min at room temperature and allowed it to stand for 24 h. Emulsification index were calculated after 24 h	E24 can display the proportion of biosurfactants produced throughout the degradation process and the capacity of stable biosurfactants to accelerate substrate breakdown and bioavailability will be boosted. Emulsifying hydrocarbon molecules consequently enhances their bioavailability	(Dusane et al. 2011) (Leite et al. 2016)
Critical micelle concentration (CMC)	The ring method was used to measure surface tension using a DuNouytensiometer at room temperature. Micelle formation occur at particular concentration of biosurfactants known as CMC. With the help of distilled water various concentration (0-500 mg/L) of extracted biosurfactant was formulated and the surface tension was recorded	Biosurfactant's effectiveness is determined by how well it dissolves in aqueous solutions. A specific quantity of biosurfactant is necessary to reduce surface tension to a minimum level, and this amount is related to the CMC value; the lower the CMC value, the process will be more efficient	(Sriram et al. 2011) (Sambanthamoorthy et al. 2014)
Penetration method	96 well ELISA microplates were taken. Hydrophobic paste containing oil and silica gel was prepared and its 200 µL were added to the wells. Subsequently, biosurfactant activity was measured by taking 10 µL of crude oil followed by addition of 90 µL culture supernatant with 10 µL of safranin solution	The presence of biosurfactants significantly speeds up silica gel's transition from the hydrophobic paste to the hydrophilic phase, resulting in a change in colour	(Walter et al. 2010) (Kumar et al. 2017)
Oil coated agar plate method	Oil coating was applied over the surface of nutrient agar media plate. Followed by streaking of given isolated strain and left the plates for incubation for 7 days at 37°C. The presence of emulsification halo around culture growth plate shows the activity of biosurfactants	A straightforward photometrical assay for determining hydrophobicity of bacteria showing bacteria's capacity to cling to hydrocarbons is a property shared by microorganisms that produce biosurfactants	(Rosenberg et al. 1980) (Burd and Ward 1996) (Shoeb et al. 2015)
Victoria Pure Blue BO	Microtitre plates coated with VPBO was prepared using VPBO (0.1mg/ml) in isopropanol solution. The isopropanol gets evaporated and NaOH solution is added to each well and incubated for 10 minutes at room temperature. The plate was dried after the aspiration of NaOH. The culture supernatantswere loaded in assay plate, sealed and incubated for 1 h. In a fresh clean 96 well microplate, VPBO dependent absorbance were measured at 625 nm. Based on reference, the value of the biosurfactant are determined.	This assay is the direct quantification of biosurfactant and it doesn't require any extraction steps. This technique offers wide aspects for comparative determination of different culture conditions for biosurfactantproduction and represents high throughput screening of biosurfactant producing microbial strains.	(Kubicki et al. 2020)

Combining several screening techniques is necessary for the effective and simultaneous identification of microbial biosurfactants (Satpute et al. 2008). For the primary screening drop collapse method, the oil displacement method and hemolytic method can be used. The drop collapse method is a straightforward and practical approach that provides a delicate yet quick manner of producing biosurfactants (Jain et al. 1991). Additionally, it enables evaluation of the presence of biosurfactants on a qualitative and quantitative level (Płaza et al. 2006). The oil displacement test is the most widely used technique for quick and simple preliminary detection of bacteria that produce biosurfactants (Płaza et al. 2006). Additionally, it can be used when the biosurfactant activity and quantity are limited (Walter et al. 2010). Previous investigations have shown the efficacy and dependability of this approach (Huy et al. 1999, Youssef et al. 2004). The hemolytic test is not a very accurate approach for biosurfactant production since some research demonstrates that it eliminated many good biosurfactant producers and, in some reports, strains with positive hemolytic activity were found to be ineffective for the production of biosurfactants (Thavasi and Jayalakshmi 2003). The most widely used technique for quantifying biosurfactant production is the emulsification index. Theoretically, a thicker emulsion layer will signify a greater production of biosurfactants. Moreover, the E24 can display the proportion of biosurfactants produced throughout the degradation process. The capacity of stable biosurfactants to accelerate substrate breakdown and bioavailability will be boosted. The deterioration rate can thus be increased (Leite et al. 2016). According to CMC, a biosurfactant's effectiveness is determined by how well it dissolves in aqueous solutions. Surface tension reduction to a minimal level requires a certain amount of biosurfactant that is proportional to the CMC value, and the lower the CMC value, the more effective the process will be (Sriram et al. 2011). Many researchers performed lipase assay for microbial biosurfactant production, the isolate confirmed lipase production on the Tributyrin Agar (TBA) plate by displaying the zone of clearing (Patel et al. 2021b, Zarinviarsagh et al. 2017). The penetration method is based on the observation that the presence of biosurfactants for determining the hydrophobicity of bacteria and is also known as the bacterial adherence to hydrocarbons significantly speeds up silica gel's transition from the hydrophobic paste to the hydrophilic phase, resulting in a color change (Walter et al. 2010). Unlike other isolates, the biosurfactant-free supernatant becomes cloudy while still being red (Hussain et al. 2021). In the oil-coated agar plate approach, bacteria's capacity to cling to hydrocarbons is a property shared by microorganisms that produce biosurfactants (Shoeb et al. 2015).

## Large scale production of biosurfactants

Surfactants hold a huge market demand worldwide. But the use of synthetic surfactants imposes harmful effects on both human life as well as imbalance the ecosystem because of their non-biodegradable nature. So, it becomes more important to focus on naturally occurring green substances to overcome this existing problem. Biosurfactants derived from microbes have solved this problem. Because they are eco-friendly and biodegradable, biosurfactants show many properties such as heterogeneity, substrate specificity, and biodegradability which has gotten a lot of attention from researchers. Above mentioned properties motivated towards their large-scale production globally (Gaur and Manickam 2021). The key factors that influence their production on a large scale are the growing organism, the substrate utilized, downstream processing, and financial inputs. Many fermentation processes are used to produce biosurfactants on a larger scale (Kronemberger et al. 2010). The fermentation procedure was carried out in a bench-size bioreactor by Joshi and Desai et al. for the manufacture of biosurfactants by *Bacillus sp*. Within 10–12 h of fermentation, the maximum concentration of biosurfactants was generated at 70–100 CMD (Critical micelle dilution) when given parameters such as initial dissolved oxygen (DO) that was set to 100% saturation and aeration rate kept at 1.0 vvm airflow (Joshi and Desai 2013). Amani et al. (2010) provided the volumetric oxygen transfer coefficient (kLa) for scaling up the production of biosurfactants, which aided in assessing increased productivity at the commercial level. In contrast to shaking flasks, they were able to boost biosurfactant production from *Bacillus subtilis* by 28% (Amani et al. 2010). Cavalcanti et al. addressed the large-scale production of biosurfactants by *Bacillus invictae* UCP 1617 (Cavalcanti et al. 2020). To demonstrate the interaction between various production circumstances and the response variable in a 5 L bioreactor, they used a complete 23-factorial design. By using this approach, the production of biosurfactants becomes 1 g/L in 72 hours, while their introduction into a 50 L bioreactor resulted in a 72-h yield of 2.42 + 1.1 g/L. Upgrading the culture medium using a response surface approach will enable strain *Wickerhamomyces anomalus* CCMA03558 to produce biosurfactants at a larger scale. By creating a pre-optimized medium that was tested in flasks and bioreactors, batch culture was employed to produce biosurfactants. The results of this experiment showed that the surface tension of the biosurfactants generated in the bioreactor and flask, respectively, decreased to 29.3 and 31.4 mN/m from 49.0 mN/m. After 24 h of fermentation, the combination of the largest microbial load and the lowest surface tension value was achieved in a 5 L bioreactor. The production of biosurfactants associated with growth is influenced by both of the previously mentioned factors (Souza et al. 2018). For the large-scale manufacture of rhamnolipid from the bacterium *Pseudomonas aeruginosa*, a bench-scale bioreactor has shown to be highly helpful. This study examined the rate of production and dependence of microbial growth on oxygen by developing a non-dispersive oxygenation apparatus under controlled conditions. When fermentation was carried out using a 1 mg/L oxygen set point, it was noted that the synthesis of glycerol and biosurfactants was consumed less (de Kronemberger et al. 2007). This approach produced 15.0 mg/L/h of rhamnolipids, which increased to 2.0 g/L/h following 7 days of fermentation. Remarkably, it was discovered that the alteration in oxygen concentration did not affect the rate of consumption, supporting the idea that the oxygenation process can enhance the efficiency of biosurfactants. The specific oxygen absorption rate increases along with the exponential microbiological growth. This proves that oxygen is crucial for the development of microbes (de Kronemberger et al. 2007).

**Figure 3.**
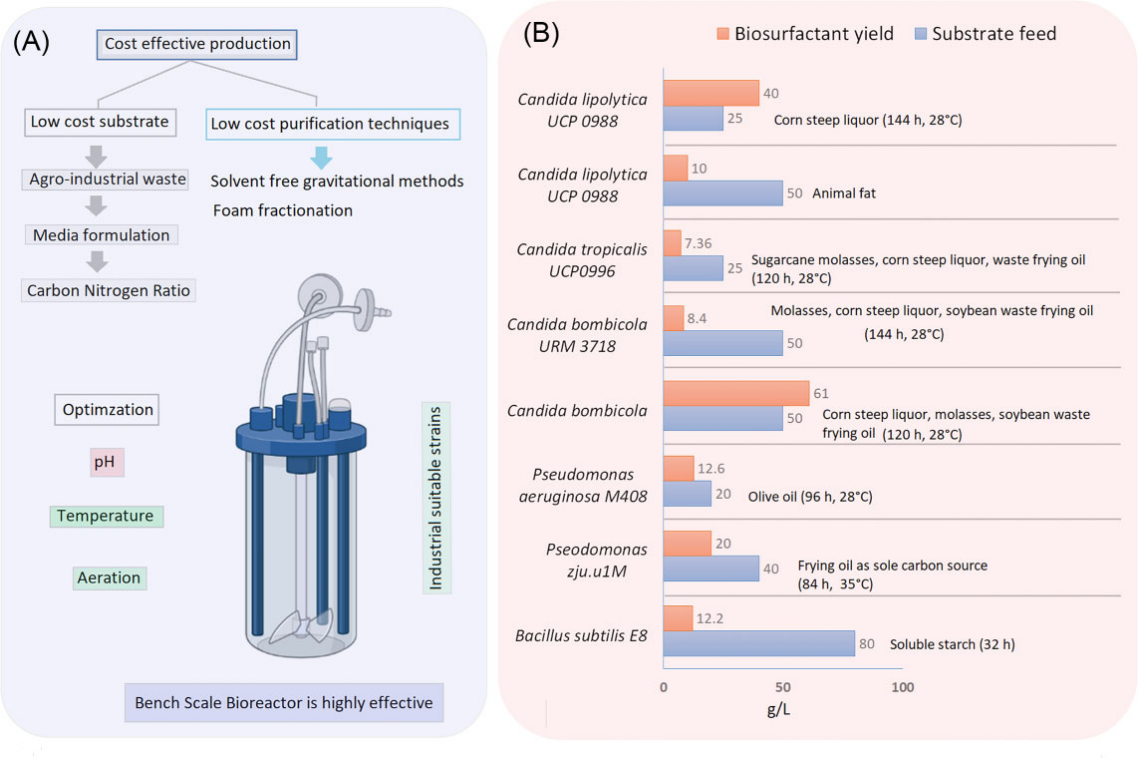
**(A)** Large scale production of biosurfactant and its major two cost effective parameters, process optimization with suitable industrial strains. **(B)** Some of the important microbial strains produced maximum yield of biosurfactant in respect to specific substrate utilization under specific time and temperature (data obtained from Singh et al. 2019).

In another study, response surface methodology (RSM) and genetic algorithms (GA) were used to build a numerical model by mathematically analyzing the variables and their interactions with the production statistics (Liyana-Pathirana and Shahidi 2005, Patel et al. 2021a). It has been reported that scaling up the production of biosurfactants from *Bacillus amyloliquefaciens* SK27 is achieved by combining the method of employing RSM and GA under optimal fermentation conditions. According to a report, the value recorded by RSM and the optimal activity of the oil displacement zone by the GA analyzer were determined to be extremely similar. The output of biosurfactants is boosted by 1.2 times using this amalgamation technique (Malik and Kerkar 2019). A cost-limiting stage makes up 60% of the entire manufacturing cost in addition to downstream processing (such as production, separation, and extraction procedures) (Chen et al. 2021). To obtain biosurfactants of high purity combinations of techniques can be used among different methods such as acid precipitation, salt precipitation, chromatography, ultra-filtration, gravity separation, and solvent extraction (Jimoh and Lin 2019). Furthermore, it was studied that the choice of extraction method plays a very important role to obtain biosurfactants of high purity as different extraction methods give different purity rates. Other factors that should be optimized while considering large-scale production of biosurfactants are pH, temperature, carbon-nitrogen ratio, and aeration (Gaur and Manickam 2021). Singh et al. (2019) described some of the important biosurfactant-producing strains with the maximum yield at a specific amount of substrates under optimum temperature that have been depicted in Fig. 3.

## Challenges of biosurfactant production

Although biosurfactants are superior to synthetic surfactants in many ways, they are still unable to compete on a commercial level because of highproduction costs and low yields (Olasanmi and Thring 2018). According to the report presented by Hazra, the production cost of biosurfactants is 20%–30% higher than that of synthetic surfactants, causing issues with large-scale production (Hazra et al. 2011). The high production cost of biosurfactants is mainly due to two main reasons. The first is related to the high cost of the substrate used, which varies from 10–30% (Sobrinho et al. 2013) to 50% (Luna et al. 2015) of the final cost of production. The second is related to the production process, which involves the purification step and accounts for the high production cost, which ranges from 60% (Freitas et al. 2016) to 70%–80% (Santos et al. 2016). Because of these reasons, researchers need to focus on reducing the production cost, which can be done by using gravity separation methods that involve biosurfactant recovery by separating the surfactant-rich phase from the fermentation broth (Makkar and Cameotra 1999) and foam fractionation, in which a solvent-free method is used for the separation of biosurfactants from the culture medium (Santos et al. 2016). On the other hand, low substrate materials, such as waste from agro-industries, which are rich in carbohydrates and have high lipid content, make substrates extremely useful and reasonably priced for the synthesis of biosurfactants (Joshi et al. 2008). This will lead to lower overall manufacturing costs and more cost-effective scaling up (Banat et al. 2010). There is a statement by Brockhaus, i.e. ‘sustainability is only sustainable when it is profitable,’ which helps in understanding the need for economic feasibility in biosurfactant production (Brockhaus et al. 2017).

## Conclusion and Future perspectives

Surfactants play a very important role in various industries worldwide, but their chemical origin imposes hazardous consequences for sustainable development. Biosurfactants are green compounds that are derived from microbes and have proven to be the best alternative to chemically produced surfactants. These have many beneficial properties, including foaming potential, thermos-resistance, low toxicity, biodegradability, and the ability to withstand extreme pH and temperature. These properties, when combined, are extremely beneficial in their widespread applications, attracting the attention of many researchers. One of the major applications is in the cleaning of the environment via hydrocarbon bioremediation, as they grow on hydrophobic surfaces, thereby augmenting the nutrient uptake of the hydrophobic substrate. There are different techniques employed for screening biosurfactants, such as the drop collapse method, emulsification index, hemolytic activity, and many more. Understanding the biosynthesis pathway of microbes associated with biosurfactant production can help optimize techniques used for screening and extraction purposes.

The production cost of biosurfactants is much higher than that of chemical surfactants. which is a major limitation associated with biosurfactants. So, there is a need to explore new techniques or optimize old ones. Further attention is needed so that researchers can work on reducing the downstream production costs and increasing their yield. Several methods, such as salt precipitation, acid precipitation, chromatography, gravity separation, ultrafiltration, and solvent extraction, can be used to produce biosurfactants of high purity. For large scales of biosurfactants, optimization of other factors like carbon-nitrogen ratio, pH, temperature, and aeration is required. Moreover, researchers need to focus on the discovery of novel strains that can grow on cost effective substrates.

## Consent to participate

Not applicable.
